# Book Review: Technology and the Psychology of Second Language Learners and Users (New Language Learning and Teaching Environments)

**DOI:** 10.3389/fpsyg.2021.619887

**Published:** 2021-03-30

**Authors:** Yang Chunhong

**Affiliations:** Department of Foreign Languages, College of Liberal Arts, Nanjing University of Information Science and Technology, Nanjing, China

**Keywords:** technology, psychology, second language learners, second language users, motivation

Technology, which has seen a widespread integration into language learning and teaching process, is often believed to have a direct influence on the cognitive and psychological processes of language learners. Its influence on such processes is more complex than perceived for both language teachers and learners (Stockwell, 2013). Despite the rich literature on the implementation of technology inside and outside the language classroom, there is still a lack of comprehensive research on its effect on the psychological processes of language learners. Edited by Mark R. Freiermuth and Nourollah Zarrinabadi, *Technology and the Psychology of Second Language Learners and Users* aims to tackle this unexamined field and explores the tripartite melding of technology, second-language learners and users, and their psychological state by reporting the latest research conducted in different technology-based learning and teaching environments.

Drawing on these studies across a number of countries, this monograph covers a wide range of different themes: processing and pragmatics, emotional and behavioral constructs, language learner identity, attitudes and perceptions, and motivation and willingness to communicate. This edited volume could not have been more well-timed in that technology-based language learning and teaching have come to the fore in recent years. In particular, online learning and teaching have become necessarily prevalent amid Covid 19 throughout the world.

In the first part, Freiermuth makes an overview of the ineluctable confluence of technology, psychology, and second-language learners and users, highlighting several critical issues such as language learner psychology, various kinds of assets, and influences of technology, which sets the scene for the following parts. Regarding language learner psychology, Freiermuth argues that it can be summarized as individual differences which will be displayed in the studies reported in this volume. The final section of this part outlines researchers from 12 different countries and their contributions to this volume based on their interests and provides a summary of the studies presented in the following chapters.

Part 2 to Part 6 report both large-scale research and case studies by scholars in different educational environments. The technologies employed in these studies include avatars, computer-assisted vocabulary acquisition software, YouTube, computer-assisted language testing program, blog, Facebook, flipping class, text-based synchronous chat, online social network, and AI. Across 15 articles, the scholars mainly explore two interconnected issues: the effect of technology application on language learners' psychology and the influence of learners' psychological state on language learning incorporating technology from different angles.

Most of the studies report positive influences of technology. The study by Karina in chapter 2 investigates how a virtual environment affects the overall acceptability of Spanish language learners' pragmatic production with the help of two types of input instruction, showing that students getting help from avatars performed best and virtual environments help contribute to their mental processes in terms of pragmatic development. Chapter 7 reports a study that addresses students' engagement in an ESP flipped course with the Facebook platform in Vietnam. The findings indicate that the integration of technology in courses increases learners' motivation, confidence and engagement in language learning, and lower cognitive resistive barriers. The study reported in chapter 13 indicates that using online social networks can change students' self-concept positively and produce psychological benefits such as certainty and positivity and affective quality. Lastly, chapter 20 examines the influence of the digital storytelling project on the motivation change of children. The data indicate that this project motivates language learners both intrinsically and extrinsically.

Nonetheless, just as the editors state in the concluding chapter, the positive influences “need to be balanced by the negative outcomes learners have experienced” (Freiermuth and Zarrinabadi, 2020, P599). It is noteworthy to see the negative psychological effects of technology reported in these studies. Anxiety in the context of the online learning environment has been examined in this volume. *Gliding Across the Digital Divide with High Anxiety* by Jako Olivier in chapter 16 investigates the level of anxiety about using a computer in EAP courses, showing that some students are anxious about learning languages via technology for they know little about computer use. Gina Paschalidou in chapter 14 finds that tutor blogging does not sufficiently motivate some students to keep on writing blogs. The learners feel uncomfortable for they might lack necessary technological skills or they feel anxious and nervous under the scrutiny of their classmates and teachers. The findings of another study in chapter 15 show that the Chinese language learners have a moderately negative perception of the Pinyin Text-to-Speech System due to a lack of technological skills and support. This research shows that the language learners' use of technologies “does not reflect a good understanding of the effective use” (Lai et al., 2016, p. 41). Thus, it is necessary for learners to develop technological competencies and enhance their self-directed use of technology in language learning (Hubbard and Romeo, 2012).

Furthermore, the distraction posed by technology has been examined as well. The study in chapter 7 finds that not all the students have beneficial learning experiences in a flipped class with Facebook. Some students are dissatisfied with this kind of instruction for they might be distracted and therefore prefer the traditional way of language learning and teaching, indicating that teachers should pay attention to individual differences when incorporating technology in language teaching practice.

Regarding the influence of learners' psychological state on technology use in language learning, this volume mainly investigates how learners' attitudes, emotions, and feelings affect technology use for language learning. The study reported in Chapter 14 indicates that fear of getting negative comments from teachers and classmates and anxiety over public exposure prevent some students from participating in the blog writing activity. Chapter 17 examines Chinese students' perceptions of AI technology for EAP speaking skills, revealing that some students prefer to use AI apps in that they hold the view that the AI-ELL app is of great use in improving their spoken English proficiency. Besides, the Y1 students use apps more frequently than Y2 students for the new students feel less anxious about using these apps while speaking English.

The groundbreaking and innovative volume fills a significant gap in exploring the ways in which technology affects the psychological state of second-language learners and users. It succeeds at providing the latest research on the confluence of technology and second-language learners' psychology and presenting many pioneering and original initiatives of using technology in different educational environments. Chapter 13 examines the relatively unexplored field and investigates the effect of TALL on adult Iranian learners' FL self-concept. In addition, chapter 16 presents research on the digital divide in developing countries and situation-specific anxiety in technology-based environments and enriches the existing literature.

It would be more comprehensive if the geographical range of studies could extend to more countries so that the audience could gain a panorama of technology integration in language learning throughout the world. In addition, it could be more significant if more studies related to the impact of online learning and teaching on learners' psychological state are included in this volume so that the language practitioner can gain insights into how to maximize teaching efficacy during and after the Covid-19 pandemic. Furthermore, it could be more important if longitudinal studies related to the changes of language learners' psychological state over time are explored so that the audience can have a better understanding of individual differences.

The book is of pedagogical value for language teachers. Just as the editors state in the concluding remarks, it is the teachers who decide how to use selected technologies. Technology is regarded as useful assistance only when language teachers incorporate it into language teaching practice to take the most advantage of its benefits for their students. The teachers can gain guidance and practical implications from the studies. Many chapters present detailed analyses of how to use technology and provide a good reference for teachers to implement technology-based classes. Furthermore, chapter 22 proposes the criteria for motivational technology-enhanced language learning activities. Besides, this volume enables teachers to realize their significant roles in technology integration and to take individual differences and needs into consideration so that technology can promote learners' language acquisition and development. In addition, the teachers need to provide support to students in the use of technologies for access to technologies alone is not sufficient.

To conclude, the research in this monograph tackles the issue of how the use of technology influences behavior and the psychological process of language learners and users. It provides some starting points and offers conceptual and practical tools for other researchers to investigate the affordances and complexities of technology-mediated language learning environments in that each study offers a literature review of relevant issues and detailed study procedures and points out future directions. The studies reported in the book have demonstrated the most up-to-date research and opened up a research niche that should not be neglected. This book could inspire researchers to find valuable research directions. In addition, this book is of great significance for second-language learners and users in that they could gain insights into the influence of technology on their mindset and behaviors and learn to use various kinds of technology appropriately to enhance motivation and positive feeling. It will attract a large audience among postgraduates, researchers, and language teachers who are interested in educational technology, language learning, and teaching and learner psychology.

## Author Contributions

YC chose the book and completed the manuscript by herself.

## Conflict of Interest

The author declares that the research was conducted in the absence of any commercial or financial relationships that could be construed as a potential conflict of interest.
